# Comparison of Macrophage Responses to African Swine Fever Viruses Reveals that the NH/P68 Strain is Associated with Enhanced Sensitivity to Type I IFN and Cytokine Responses from Classically Activated Macrophages

**DOI:** 10.3390/pathogens9030209

**Published:** 2020-03-12

**Authors:** Giulia Franzoni, Elisabetta Razzuoli, Silvia Dei Giudici, Tania Carta, Grazia Galleri, Susanna Zinellu, Mauro Ledda, Pierpaolo Angioi, Paola Modesto, Simon P. Graham, Annalisa Oggiano

**Affiliations:** 1 Department of Animal Health, Istituto Zooprofilattico Sperimentale della Sardegna, 07100 Sassari, Italy; Silvia.DeiGiudici@izs-sardegna.it (S.D.G.); tcarta@uniss.it (T.C.); Susanna.Zinellu@izs-sardegna.it (S.Z.); pierpaolo.angioi@izs-sardegna.it (P.A.); annalisa.oggiano@izs-sardegna.it (A.O.); 2 Department of Genoa, Istituto Zooprofilattico Sperimentale della Liguria, Piemonte e Valle d’Aosta, 16129 Genova, Italy; Elisabetta.Razzuoli@izsto.it (E.R.); paola.modesto@izsto.it (P.M.); 3 School of Veterinary Medicine, University of Sassari, 07100 Sassari, Italy; vetleddamauro@gmail.com; 4 Department of Clinical and Experimental Medicine, University of Sassari, 07100 Sassari, Italy; galleri@uniss.it; 5 Porcine Reproductive and Respiratory Syndrome Immunology Group, The Pirbright Institute, Pirbright GU24 0NF, UK; simon.graham@pirbright.ac.uk

**Keywords:** ASFV, monocyte-derived macrophages, polarized activation, flow cytometry, cytokines

## Abstract

African swine fever (ASF) poses a severe threat to the global pig industry for which currently there is no available vaccine. The aetiological ASF virus (ASFV) has a predilection for cells of the myeloid lineage, however little is known about its interaction with polarised macrophages. This study focused on the in vitro interactions of porcine monocyte-derived un-activated (moMΦ), classically (moM1), alternatively (moM2), and IFN-α-activated macrophages with two genotype I ASFV strains: virulent 22653/14 and attenuated NH/P68. At a high multiplicity of infection, NH/P68, but not 22653/14, presented a reduced ability to infect moM1 and IFN−α-activated moMΦ compared to moMΦ. IFN-α activation resulted in a dose-dependent reduction in the proportion of ASFV-infected cells. Both strains replicated efficiently in all the subsets. While higher levels of IL-1α, IL-1β, and IL-18 were secreted by NH/P68-infected moM1 compared to 22653/14, both strains negatively affected moMΦ ability to release IL-6, IL-12, TNF-α in response to classical activation or stimulation with a TLR2 agonist. Our results suggest that ASFV 22653/14 covertly replicates in macrophages, compromising the development of effective immune responses. Attenuated NH/P68 has partially lost these mechanisms, which may enhance immune surveillance. A better understating of these mechanisms should aid the rational design of live attenuated ASFV vaccines.

## 1. Introduction

African swine fever (ASF) is a contagious viral disease of domestic pigs and wild boar, for which there is currently no licensed vaccine or treatment available. It is currently a threat for the global swine industry, being present in many sub-Saharan African countries, the Russian Federation, Trans-Caucasus, East and Central Europe, Sardinian and Southeast Asia [1]. The aetiological agent is the African swine fever virus (ASFV), a large, enveloped double-stranded DNA virus, which is the only member of the *Asfarviridae* family [1]. A better understanding of how ASFV interacts with the porcine immune system is essential to aid the development of safe and efficacious vaccines. Macrophages are the primary target cell for ASFV and provide a first line of defense against pathogens, thus their interaction with ASFV is of major importance. As we recently reviewed, in vitro studies on macrophages showed that attenuated ASFV strains induce enhanced expression of cytokines (IFN-α, IFN-β, IL-1β, IL-12, IL-18, TNF-α) and chemokines (CCL4, CXCL8, CXCL10) compared to highly virulent strains [2,3,4,5,6,7]. Both virulent and attenuated ASFV down-regulated CD14 and CD16 expressions on infected macrophages, which may affect their anti-microbial/viral activity [6,7,8]. Overall, these studies suggest that virulent ASFV isolates have evolved mechanisms to counteract macrophage responses, whereas expression of key cytokines and chemokines from macrophages in response to attenuated ASFV strains could enhance the induction of innate immune responses and promote the induction of effective adaptive immune responses [7]. Despite this central role for macrophages in ASF immunobiology, little is known about responses of macrophages in distinct activation states to ASFV. Macrophages can be activated either ‘classically’ or ‘alternatively’, resulting in two distinct functional subsets, referred to as M1 and M2, respectively. M1 macrophages provide antimicrobial and pro-inflammatory functions, whereas M2 are associated with mechanisms of immunosuppression and wound healing [9]. In pigs, as in other species, M1 polarization can be achieved in vitro using IFN-γ and LPS, resulting in up-regulation of MHC and co-stimulatory molecules and release of pro-inflammatory cytokines, whereas M2 polarization, achieved through IL-4, is characterized by CD203a up-regulation [10,11].

We previously investigated responses of porcine monocyte-derived unactivated (moMΦ), classically (moM1), alternatively (moM2) activated macrophages with the virulent 22653/14 and attenuated BA71V ASFV strains. BA71V is completely avirulent in pigs and lacks the capacity to confer protection against homologous ASFV challenge (BA71) [12], thus in the present study the Sardinian isolate 22653/14 was compared to the attenuated NH/P68 ASFV. Immunization with NH/P68 conferred protection against homologous and heterologous ASFV challenge and this isolate can be studied to provide insights into the induction of protective immune responses [13,14]. In this work, IFN-α-activated moMΦ were also included, as a model for type I IFN activation, which are central to the innate immune response to viral infection [15]. Ability of these two ASFV strains of diverse virulence to infect, replicate, modulate phenotype and functionality of different macrophage subsets were assessed, using multi-parametric flow cytometry, quantitative (RT-)PCR and ELISA.

## 2. Results

### 2.1. Susceptibility of Monocyte-Derived Macrophage Subsets to ASFV Infection

**Figure 1:**
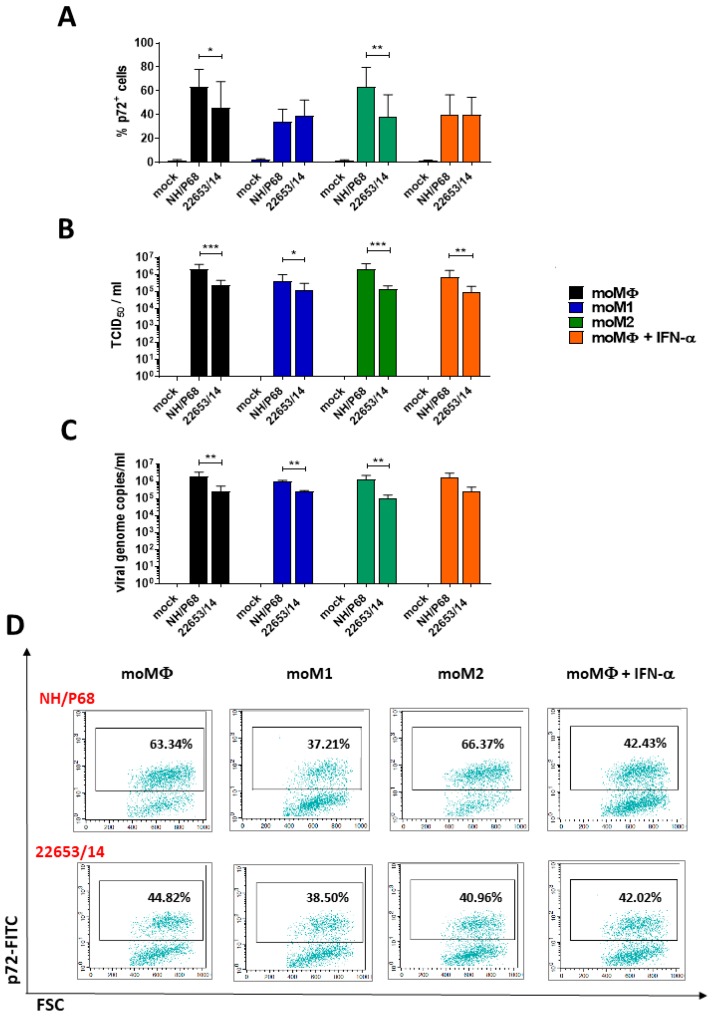
Comparison of the ability of ASFV NH/P68 and 22653/14 to infect porcine monocyte-derived macrophage subsets. moMΦ, moM1, moM2, IFN-α-activated moMΦ were infected with the low virulence NH/P68 or the virulent 22653/14 ASFV strains using a MOI of 1, alongside mock-infected controls. 21 h pi percentages of ASFV p72^+^ cells (**A**), levels of infectious viral progeny (TCID_50_/_mL_) (**B**) and genome viral copies numbers/ ml (**C**) in culture supernatants were assessed. The mean data +/− SD from five (p72, titration) or four (genome viral copies) independent experiments utilizing different animals are shown. NH/P68, and 22653/14 values were compared using a Mann-Whitney test; *** *p* < 0.001, ** *p* < 0.01, * *p* < 0.05. Representative dot plots are displayed to demonstrate the p72 labelling of macrophage subsets infected with the two ASFV strains (**D**).

The susceptibility of macrophage subsets to ASFV infection was assessed by quantification of cells containing ASFV late protein p72, viral titres and ASFV genome copy numbers in the cell culture supernatants (Figure 1). Cells were infected with the attenuated NH/P68 or the virulent Sardinian isolate 22653/14, alongside mock-infected controls. Using a multiplicity of infection (MOI) of 1, the attenuated NH/P68 infected macrophage subsets more efficiently than the virulent 22653/14: NH/P68 infection resulted in a greater amount of both viral titre and viral genome copies in culture supernatants compared to 22653/14 (Figure 1). In addition, a greater proportion of p72^+^ cells were detected 21 h post-infection (pi) with NH/P68 in both moMΦ and moM2 (Figure 1). In contrast, the proportion of p72^+^ cells detected in moM1 or moMΦ + IFN-α cultures were comparable between the attenuated NH/P68 and the virulent 22653/14 strain. However, similarly to moMΦ and moM2, there were higher levels of NH/P68 infectious particles and genomes detected in the supernatants. In order to further assess whether NH/P68 and 22653/14 replicated in macrophages with different efficiencies, we compared the mean fluorescence intensity (MFI) of p72^+^ cells, as a measure of this late viral protein load on a per cell basis. In all the subsets, there were no statistically significant differences between the MFI of p72^+^ cells infected with NH/P68 or 22653/14 (Figure S1).

We therefore may only speculate that the higher levels of NH/P68 infectious particles in moM1 or moMΦ + IFN-α supernatants compared to 22653/14 may reflect a greater efficiency of the attenuated strain to assemble and exit the macrophage.

**Figure 2:**
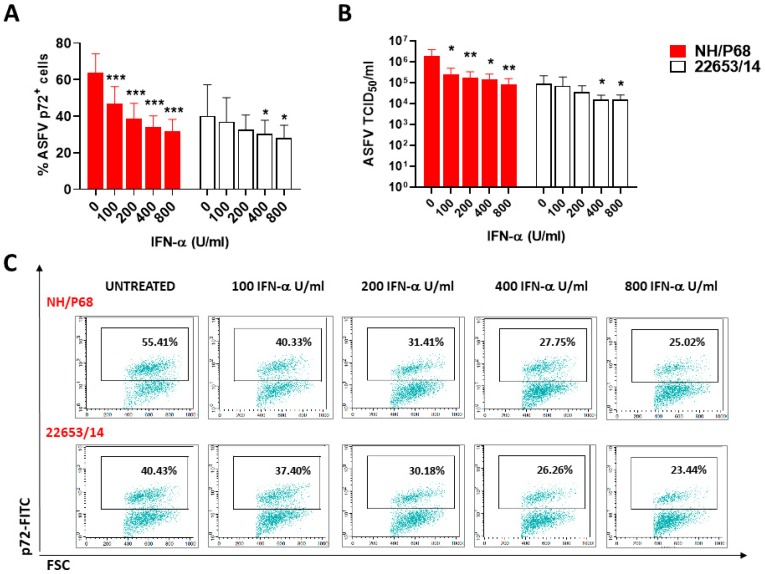
Dose dependent effect of IFN-α on ASFV susceptibility *in vitro*. moMΦ were left untreated or activated with different concentration of recombinant porcine IFN-α (100, 200, 400, 800 U/mL). After 24 h, cells were infected with the low virulence NH/P68 or the virulent 22653/14 ASFV strains, using a MOI of 1, alongside mock-infected controls. 21 h pi percentages of ASFV p72^+^ cells were evaluated using flow cytometry (**A**) and infectious viral progeny in culture supernatants were assessed by titration (TCID_50_/_mL_) (**B**). The mean data +/− SD from four independent experiments utilizing different animals are shown. For each condition (NH/P68, and 22653/14) values of IFN-α−activated moMΦ were compared to the corresponding un-treated condition (moMΦ), using a Kruskal-Wallis test followed by Dunn’s multiple comparison test; *** *p* < 0.001, ** *p* < 0.01, * *p* < 0.05. Representative dot plots are displayed to demonstrate the p72 labelling of untreated and IFN-α treated macrophages infected with the two ASFV strains (**C**).

NH/P68 presented a reduced ability to infect both moM1 and moMΦ activated with 100 U/mL of IFN-α (moMΦ + IFN-α) compared to moMΦ, whereas no statistically significant differences were observed between 22653/14-infected macrophage subsets (Figure 1). To further investigate the IFN-α induced inhibitory effect on ASFV strains of diverse virulence, moMΦ were activated with a titration of recombinant IFN-α (100, 200, 400, and 800 U/mL) and after 24 h cells were infected with NH/P68 or 22653/14. IFN-α activation resulted in a dose-dependent reduction in the proportion of ASFV-infected cells and the infectious progeny virus released (Figure 2). Only at high IFN-α doses (400 and 800 U/mL) there were significant differences observed between 22653/14 infected moMΦ and IFN-α-activated moMΦ, whereas NH/P68 was sensitive to lower IFN-α concentration (100 U/mL) (Figure 2). To determine whether virulent 22653/14 sensitivity to high doses of IFN-α was a strain-specific effect, we tested additional virulent ASFV isolates: genotype I Nu81.2, genotype II Arm07 and genotype IX Ken06.Bus. Activation of moMΦ using high IFN-α concentration (800 U/mL) resulted in reduction of the proportion of ASFV-infected cells for all the tested strains (Figure S2).

### 2.2. Kinetic Analysis of ASFV Replication in Macrophage Subsets

**Figure 3:**
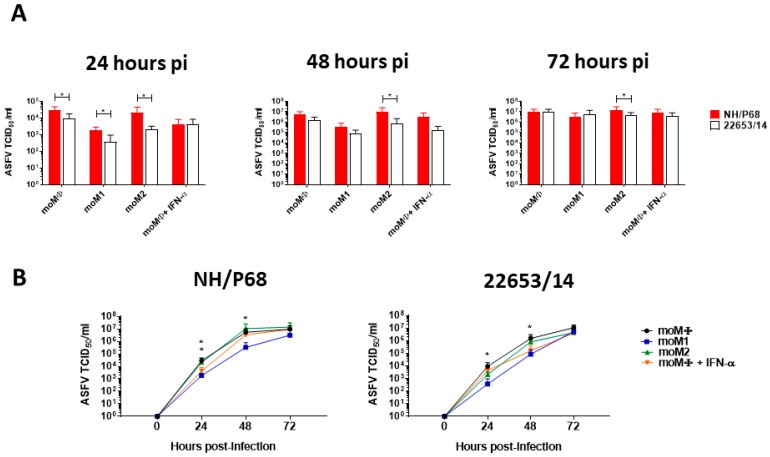
Growth kinetics of ASFV strains in macrophage subsets. moMΦ, moM1, moM2, IFN-α-activated moMΦ were infected with the low virulence NH/P68 or the virulent 22653/14 ASFV strains using an MOI of 0.01. At 0, 24, 48, 72 h pi duplicate samples were collected and infectious viral progeny in culture supernatants were assessed by titration (TCID_50_/_mL_). The mean data +/− SD from three independent experiments utilizing different animals are shown. (**A**) At each time-point and for each subset, values of NH/P68 and 22653/14 infected cells were compared using a Mann-Whitney test. (**B**) At each time-point values of activated macrophages were compared to the corresponding un-activated control (moMΦ), using a Kruskal-Wallis test followed by Dunn’s multiple comparison test; *** *p* < 0.001, ** *p* < 0.01, * *p* < 0.05.

A kinetic analysis of the infection with the attenuated NH/P68 and the virulent 22653/14 ASFV strains was performed in all the macrophage subsets using a MOI of 0.01. We opted to use a low MOI due to the fact that at 48 h pi using a MOI of 1, the majority of ASFV-infected macrophages were detached due to cell death, especially with infection with NH/P68 and at 72 h pi all ASFV-infected macrophages were dead (data not shown). Replication was measured by longitudinally assessing viral titres present in cell culture supernatants. Both isolates replicated efficiently in all macrophage subsets, but at 24 h pi the attenuated NH/P68 was more efficient than the virulent 22653/14 at replicating in moMΦ, moM1 and moM2. At later times post-infection (48, 72 h pi), no differences were observed between strains, except for higher levels of virus particles in NH/P68 compared to 22653/14-infected moM2 (Figure 3A).

Differences between macrophage subsets were also observed. For both strains, lowest titres were detected in moM1 cultures (Figure 3B). Nevertheless, at 72 h pi no significant differences in infectious viral levels between moM1 and moMΦ were detected (Figure 3B). At 24 h pi with the attenuated NH/P68, but not the virulent 22653/14, IFN-α-activated moMΦ presented lower viral levels then un-activated moMΦ (Figure 3). This was in accordance with the data obtained using a MOI of 1 (Figure 1), but no differences were detected at later time points (Figure 3B).

### 2.3. Modulation of MHC Expression by ASFV Strains of Diverse Virulence

**Figure 4:**
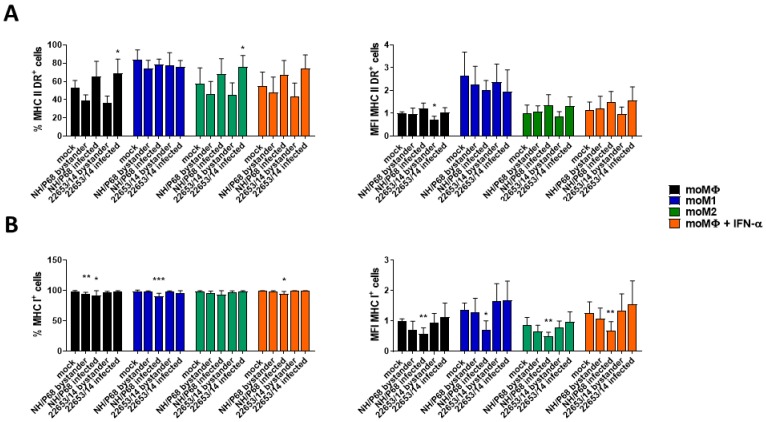
Effect of ASFV on the expression of MHC class I and II on macrophage subsets. moMΦ, moM1, moM2, IFN−α−activated moMΦ were infected with the low virulence NH/P68 or the virulent 22653/14 strains using a MOI 1, alongside mock-infected controls. 21 h pi, expression of surface expression of MHC class II DR (**A**) and MHC class I (**B**) and intracellular levels of ASFV p72 were assessed by flow cytometry. The mean data +/− SD from four independent experiments utilizing different animals are shown. For each marker, both percentages (left) and mean fluorescence intensity (MFI) (geometric mean) (right) of MHC class II DR^+^ or MHC class I^+^ cells are displayed. MFI data are presented as fold change relative to the mock-infected un-activated condition (moMΦ mock). Values of ASFV-infected or bystander macrophages were compared to the corresponding mock-infected control, using a Kruskal-Wallis test followed by Dunn’s multiple comparison test; *** *p* < 0.001, ** *p* < 0.01, * *p* < 0.05.

With the aim of further characterising the differences between ASFV strains of diverse virulence, the effect of infection on MHC class I and class II DR expression was investigated. Macrophage subsets were infected with NH/P68 and 22653/14 using a MOI of 1, alongside mock-infected controls. At 21 h pi, the expression of surface markers and intracellular levels of late viral protein p72 were assessed by flow cytometry. The gating strategy and representative dot plots are presented in Figure S3. Negligible variations in the MHC class II DR expression on mock-infected and ASFV-infected macrophage subsets were detected, but statistically significant differences were observed between infected (p72^+^) and bystander (p72^−^) moMΦ, moM2, IFN-α-activated-moMΦ, with p72^+^ cells presenting higher levels of MHC class II DR compared to bystander uninfected cells (Figure 4A). This trend was not observed with moM1, which presented a higher expression of MHC class II DR irrespective of infection. In contrast, infection with NH/P68, but not 22653/14, resulted in down-regulation of MHC class I on infected cells in all the macrophage subsets (Figure 4B). Variations were observed mainly in the MFI value of MHC I^+^ cells, and only small differences were detected between percentages of MHC I^+^ mock-infected and NH/P68-infected cells and not in all the tested pigs. Differences were also observed between infected and bystander cells, suggesting that MHC class I modulation is a direct consequence of virus infection (Figure 4B).

**Figure 5:**
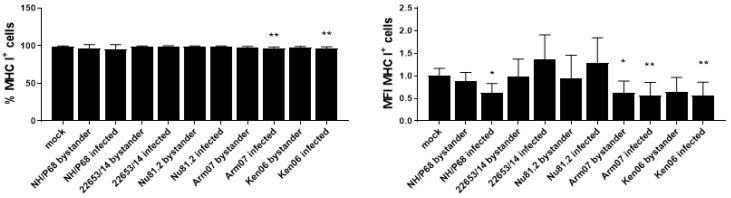
Modulation of MHC class I expression by ASFV strains of diverse genotype and virulence. moMΦ were infected with the low virulence genotype I NH/P68 or the following virulent ASFV isolates: genotype I 22653/14, genotype I Nu81.2, genotype II Arm07, genotype IX Ken.Bus06. A MOI 1 was used and mock-infected control was included in the experiment. 21 h pi, expression of surface expression of MHC class I and intracellular levels of ASFV p72 were assessed by flow cytometry. The mean data +/− SD from four independent experiments utilizing different animals are shown. For each marker, both percentages (left) and mean fluorescence intensity (MFI) (geometric mean) (right) of MHC class I^+^ cells are displayed. MFI data are presented as fold change relative to the mock-infected un-activated condition (moMΦ mock). Values of ASFV-infected or bystander macrophages were compared to the mock-infected control, using a Kruskal-Wallis test followed by Dunn’s multiple comparison test; *** *p* < 0.001, ** *p* < 0.01, * *p* < 0.05.

In our previous work we observed that the attenuated BA71V also inhibited MHC class I expression on moMΦ [6], thus we wanted to determine whether the absence of MHC class I down-regulation following 22635/14 infection was a strain-specific effect. moMΦ infected with the virulent genotype I Sardinian strains (Nu81.2 and 22653/14) presented a slightly higher MHC class I expression, albeit without statistical significance (Figure 5). Infection with both genotype II (Arm07) and genotype IX (Ken06.Bus) virulent isolates induced MHC class I down-regulation (observed mainly in the MFI value of MHC I^+^ cells), but with little differences between infected and bystander cells (Figure 5).

### 2.4. Cytokine Responses of ASFV-Infected Macrophage Subsets

**Figure 6:**
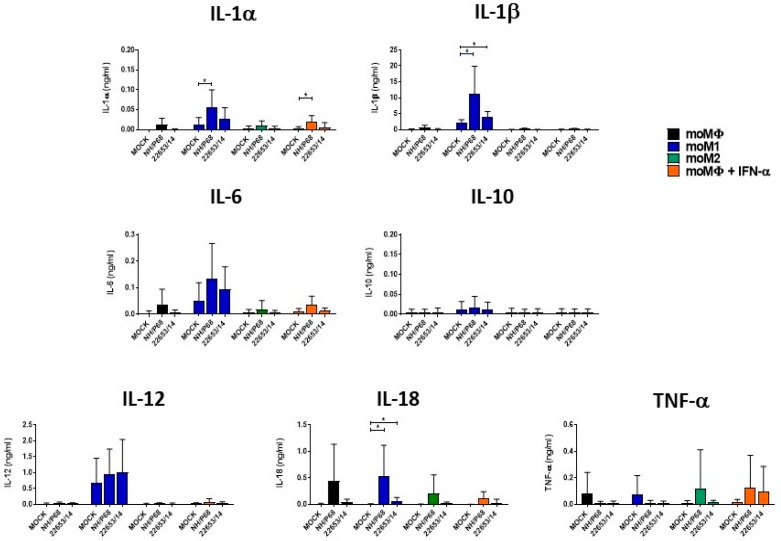
Cytokine release by macrophage subsets in response to infection with ASFV strains of diverse virulence. moMΦ were left untreated or polarised with different activators. 24 h post-activation, supernatants were removed and then moMΦ, moM1, moM2, IFN-α-activated moMΦ were infected with the low virulence NH/P68 or the virulent 22653/14 strains using a MOI 1, alongside mock-infected controls. 21 h pi, the amount of IL-1α, IL-1β, IL-6, IL-10, IL-12, IL-18, TNF-α in culture supernatants were determined using a multiplex bead-based immunoassay. The mean data +/− SD from four independent experiments utilizing different animals are shown. Values of ASFV-infected cells were compared to the corresponding mock-infected control using an unpaired t-test or a Mann-Whitney test; *** *p* < 0.001, ** *p* < 0.01, * *p* < 0.05.

**Figure 7:**
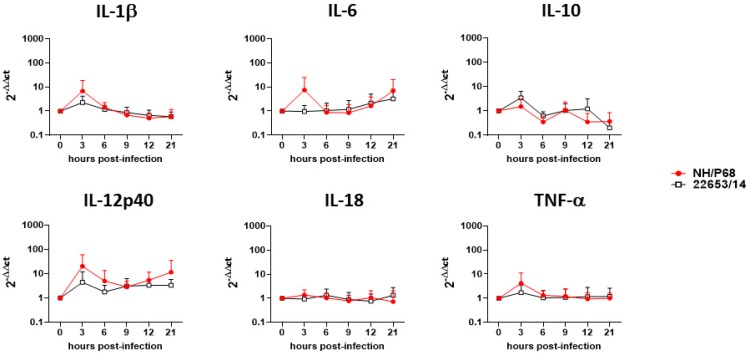
Cytokine gene expression in ASFV-infected macrophages. moMΦ were infected with the low virulence NH/P68 or the virulent 22653/14 strains using a MOI 1. 0, 3, 6, 9, 12, 21 h pi, gene expression of IL-1β, IL-6, IL-10, IL-12p40, IL-18, TNF-α were determined using qPCR. Data were normalized on the values of un-infected control (0 h pi), and expressed as ΔΔCt, where ΔΔCt = (ΔCt observed in un-infected moMΦ) − (ΔCt observed in ASFV-infected moMΦ). The mean data +/− SD from six independent experiments utilizing different animals are shown. Values of ASFV-infected cells were compared to the corresponding mock-infected control using a Kruskal-Wallis test followed by Dunn’s multiple comparison test; *** *p* < 0.001, ** *p* < 0.01, * *p* < 0.05.

Cytokine responses of monocyte-derived macrophage subsets to NH/P68 and 22653/14 infection were next investigated. Cells were infected using a MOI of 1, alongside mock-infected controls, and 21 h pi cytokine levels in culture supernatants were quantified. Higher levels of IL-1α, IL-1β, IL-18 and IL-6 were observed from NH/P68-infected moM1 compared to the virulent 22653/14 and the mock-infected control, although the latter was not statistically significant (Figure 6). None of the tested cytokines (IL-1α, IL-1β, IL-6, IL-10, IL-12, IL-18 and TNF-α) were released in response to ASFV by the other macrophage subsets, with the exception of IL-1α from IFN-α−activated moMΦ and IL-18 from both moM2 and IFN-α-activated moMΦ, although the latter two were not statistically significant (Figure 6). A small increase in the IL-18 levels in culture supernatants was also observed in NH/P68-infected moMΦ from one of the tested pigs, but no release was detected from the other pigs analyzed. Since studies had reported that NH/P68 induced enhanced cytokine gene expression or production in macrophages compared to a virulent isolate [2,3], cytokine gene (IL-1β, IL-6, IL-10, IL-12p40, IL-18, TNF-α) expression in moMΦ after NH/68 or 22653/14 infection were monitored over-time. At 3 h pi, an enhanced expression of IL-1β, IL-6, and TNF-α was observed in NH/P68-infected compared to 22653/14-infected macrophages, although it was not statistically significant (Figure 7). On the contrary, infection with both strains resulted in a reduction in the IL-10 gene expression at later time pi (Figure 7).

### 2.5. Effect of ASFV Infection on Ability to Respond to C1assical Activation or Stimulation with TLR2 Agonist

**Figure 8:**
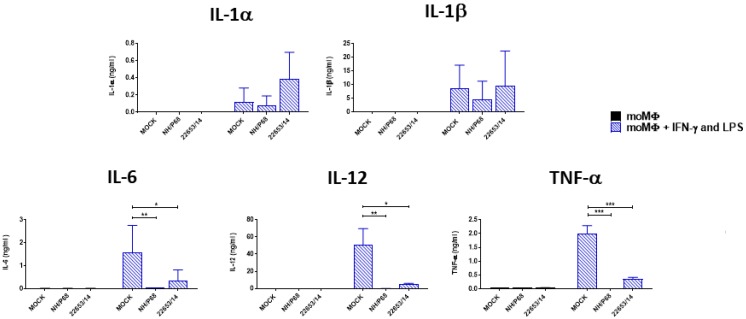
Effect of infection with ASFV strains of diverse virulence on macrophage response to classical activation. moMΦ were infected with the low virulence NH/P68 or the virulent 22653/14 strains using a MOI 1, alongside mock-infected controls. 21 h pi, cells were activated using recombinant porcine IFN-γ and LPS. 24 h later, the amount of IL-1α, IL-1β, IL-6, IL-12, TNF-α in culture supernatants were determined using a multiplex bead-based immunoassay. The mean data +/− SD from three independent experiments utilizing different animals are shown. Values of ASFV-infected cells were compared to the corresponding mock-infected control using an unpaired t-test or a Mann-Whitney test; *** *p* < 0.001, ** *p* < 0.01, * *p* < 0.05.

**Figure 9:**
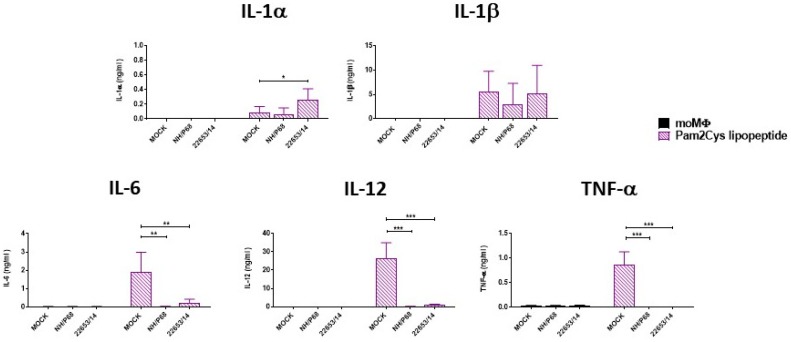
Effect of infection with ASFV strains of diverse virulence on macrophage response to TLR2 agonist. moMΦ were infected with the low virulence NH/P68 or the virulent 22653/14 strains using a MOI 1, alongside mock-infected controls. 21 h pi, cells were activated using a Pam2Cys lipopeptide (1 μg/mL). 24 h later, the amount of IL-1α, IL-1β, IL-6, IL-12, TNF-α in culture supernatants were determined using a multiplex bead-based immunoassay. The mean data +/− SD from three independent experiments utilizing different animals are shown. Values of ASFV-infected cells were compared to the corresponding mock-infected control using an unpaired t-test or a Mann-Whitney test; *** *p* < 0.001, ** *p* < 0.01, * *p* < 0.05. Effect of infection with ASFV strains of diverse virulence on macrophage response to TLR2 agonist. moMΦ were infected with the low virulence NH/P68 or the virulent 22653/14 strains using a MOI 1, alongside mock-infected controls. 21 h pi, cells were activated using a Pam2Cys lipopeptide (1 μg/mL). 24 h later, the amount of IL-1α, IL-1β, IL-6, IL-12, TNF-α in culture supernatants were determined using a multiplex bead-based immunoassay. The mean data +/− SD from three independent experiments utilizing different animals are shown. Values of ASFV-infected cells were compared to the corresponding mock-infected control using an unpaired t-test or a Mann-Whitney test; *** *p* < 0.001, ** *p* < 0.01, * *p* < 0.05.

We finally investigated the effect of ASFV infection on the ability of moMΦ to respond to external stimuli. First, the effect of either attenuated or virulent ASFV strains on the ability of moMΦ to release cytokines in response to classical activation (IFN-γ and LPS) was assessed (Figure 8). moMΦ were infected with NH/P68 or 22653/14 using a MOI of 1, alongside mock-infected controls, and after 21 h pi cells were activated with IFN-γ and LPS. 24 h post-activation, cytokine levels in culture supernatants were determined. ASFV infection markedly reduced the ability of moMΦ to release IL-6, IL-12 and TNF-α in response to classical stimulation (Figure 8). Finally, the effect of ASFV infection on the ability of moMΦ to respond to another stressor was investigated. 21 h post-infection, cells were activated with a TLR2 agonist (Pam2Cys lipopeptide). Infection with either NH/P68 and 22653/14 ASFV resulted in a reduced ability of moMΦ to release the same cytokines (IL-6, IL-12, TNF-α) in response to stimulation with Pam2Cys lipopeptide (Figure 9).

## 3. Discussion

ASFV infects immune cells of the myeloid lineage, with macrophages considered its primary target cells, nevertheless few studies have analyzed ASFV interactions with activated macrophages. Macrophages are a heterogenous population and present remarkable plasticity. *In vivo*, they modify their phenotype and function in response to environmental signals. M1 and M2 are not ontogenetically defined but are thought to represent the two extremes of diverse functional activation status [16]. We hypothesized that macrophages in antithetic polarized states might respond differently to ASFV, with additional differences between attenuated and virulent strains. Information obtained might help to elucidate virological and cellular elements important for ASFV interaction with its main target cell type, which may allow a better understanding of mechanisms underlying the development of pathological or protective immune responses [7].

We first focused on determining macrophage subsets susceptibility to ASFV infection with attenuated NH/P68 or virulent 22653/14. Both strains infected all subsets tested, but infection of macrophages with NH/P68 resulted in higher virus titers and higher viral genome copies in culture supernatants compared to the virulent 22653/14. A similar trend was observed in previous studies on monocytes, monocyte-derived dendritic cells (moDC) [17], monocyte-derived macrophages [3], porcine alveolar macrophages (PAM) and another porcine cell line (WSL) [18], suggesting that this attenuated strain possess mechanisms to more efficiently replicate in myeloid cells, at least *in vitro*. Differences between macrophage subsets were also observed. NH/P68 presented a slightly reduced ability to infect both moM1 and moMΦ activated with 100 U/mL of IFN-α compared to moMΦ. These results are in accordance with a previous study, where researchers observed that several virulent isolates (BA71, Georgia 2007/1, OUR T88/1) presented a greater ability to infect IFN-α-activated (2000 U/mL) PAM compared to the attenuated strain OUR T88/3, and differences were linked to genes within multigene families 360 and 505 [19]. Using high doses of IFN-α (800 U/mL), we observed a reduction in the proportion of infected cells also for virulent ASFV isolates, independently of the genotype. These differences might be linked to the distinct macrophage populations used in the two studies (monocyte-derived MΦ versus PAM), which might present variations in their response to IFN-α.

We next performed a kinetic analysis of ASFV replication in macrophage subsets. Both ASFV strains efficiently replicated in all macrophage subsets. In our previous work, we observed that the avirulent BA71V was unable to efficiently replicate in macrophages [6], but this was probably linked to its adaptation to grow in VERO cells and this might explain its inability to induce protective immune response in pigs. In that study, we also observed that moM2 displayed slightly higher permissiveness to BA71V replication compared to moMΦ and moM1, suggesting that alternative macrophage activation was correlated with increased susceptibility to attenuated ASFV [6]. In this work, no significant differences were observed between NH/P68-infected moMΦ and moM2, but at 48- and 72-h pi differences between strains were observed only in moM2, with higher levels of viral particles detected NH/68 compared to 22653/14-infected moM2. These data suggest that ASFV initially infected moM1 with a lower intensity compared to the other subsets, but the virus was able to overcome cellular defense mechanisms and replicate efficiently in this polarized subset. Our results are similar to those reported by Dutry et al., where it was observed that human macrophages activated with IL-4 were more susceptible to infection with highly pathogenic avian influenza virus (H5N1) compared to IFN-γ activated macrophages, but at later time points post-infection no differences were detected in the viral titers of culture supernatants of the two macrophage subsets [20].

Modulation of macrophage expression of MHC class I and II might affect antigen-presentation and development of protective T cell immune responses. Infection with the attenuated NH/P68 but not the virulent 22653/14 resulted in down-regulation of MHC class I on infected macrophages. We observed similar results in BA71V-infected moMΦ and in moDC infected with attenuated but not virulent ASFV strains [6,17] and, as we previously speculated, MHC class I down-regulation on antigen-presenting cells might result in NK cell activation in vivo [7]. In fact, a previous in vivo study reported a correlation between NK activation and protection in pigs inoculated with NH/P68 and then challenged with the homologous virulent L60 [13]. Interestingly, we found that infection with virulent genotype I ASFV isolates (Nu81.2 and 22653/14) did not reduce MHC class I expression on macrophages, whereas, infection with virulent isolates belonging to genotype II (Arm07) or IX (Ken06.Bus) down-regulated MHC class I expression with intensity similar or even higher than NH/P68. Our results are similar to those observed by Arav, where infection of bone marrow-derived macrophages (BMDM) with the virulent genotype VIII Malawi Lil20 resulted in lower MHC I expression compared to that of the virulent genotype I Benin 97/1, but similar to that of attenuated NH/P68 [21]. Interestingly, it was observed that deletion of the gene coding for CD2v, whose ORF is interrupted in NH/P68 [22], from Benin 97/1 resulted in lower MHC I expression on BMDM, to level similar to that of NH/P68. Deletion of CD2v from Malawi Lil20 did not influence MHC I expression [21]. Overall, these results suggest that CD2v might represent a more relevant virulence factor for genotype I ASFV isolates compared to ASFV isolates belonging to different genotype, as previously speculated by others [12]. Infection with either virulent or attenuated ASFV strains had negligible effects on MHC class II DR expression on all the macrophage subsets, in accordance with previous studies [7]. Higher expression of MHC class II DR was observed in ASFV-infected compared to bystander moMΦ, moM2 and IFN-α-activated moMΦ and this might be linked to the predilection of ASFV to cells with a more mature phenotype, in accordance with a previous study [8]. In fact, this trend was not observed with moM1, which presented a higher expression of MHC class II DR, irrespective of infection.

In our previous work, we reported a higher release of IL-1α, IL-1β and IL-18 from BA71V-infected moM1 compared to 22653/14 [6] and in this study we observed higher release of the same cytokines from NH/P68-infected moM1 compared to the virulent Sardinian isolate and mock-infected controls. IL-18 is a potent inducer of IFN-γ [23], which might contribute to the development of a protective cellular immune response against ASFV, thus virulent ASFV isolates might have developed mechanisms to counteract its release. Enhanced levels of the pro-apoptotic IL-1β were also detected in NH/P68-infected moM1, and as we previously speculated for BA71V, this might result in apoptosis of bystander cells, limiting in vivo ASFV replication and spread [6]. Our results highlighted that cytokine release in response to ASFV was performed mainly by moM1, and negligible release of all the tested cytokines by moMΦ, moM2, IFN-α-activated moMΦ was detected. Further studies in moM1 should be performed, in order to investigate differences between strains of diverse virulence, aiming at understanding which viral factors, lost in attenuated strains, inhibit release of these key cytokines. In contrast to our results, previous studies reported that infection of un-activated macrophages with attenuated ASFV strains results in enhanced expression of several key regulatory cytokines compared to virulent strains (IFN-α, IFN-β, TNF-α, IL-6, IL-12p40, IL-15) [2,3,4], thus in this study cytokine gene expression (IL-1β, IL-6, IL-12p40, IL-18, TNF-α) in ASFV-infected moMΦ was also monitored over time. In accordance with Gil et al. (2003, 2008) [2,3], 3 h pi an enhanced expression of IL-1β, IL-6, and TNF-α was observed in NH/P68-infected compared to 22653/14-infected moMΦ, although no increase in the levels of these cytokines were detected in culture supernatants of NH/P68-infected moMΦ. Post-transcriptional mechanisms might disturb synthesis and/or secretion of these cytokines. Induction of the anti-inflammatory IL-10 was also monitored and we observed that ASFV infection resulted in reduced gene expression of this cytokine at later time pi, with no differences between strains.

Finally, we assessed whether ASFV infection inhibited M1 activation of macrophages. Since 22653/14 did not induce any cytokine release from moM1, whereas IL-1α, IL-1β, IL-18 were detected in culture supernatants of both NH/P68 and BA71V infected moM1 (Figure 6 of this study, [6]), we speculated that the virulent 22653/14 ASFV may have evolved mechanisms to compromise the macrophage’s ability to respond to classical activation. In addition, previous studies reported that ASFV infection resulted in down-regulation of CD14 and CD16, potentially compromising macrophage anti-microbial and antiviral activity [7]. Our results clearly show that ASFV infection inhibited the ability of macrophages to respond to external stimuli, with marked reduction in their ability to release IL-6, IL-12 and TNF-α in response to stimulation with IFN-γ and LPS or a TLR2 agonist. At early time post infection (2 h pi), neither virulent nor attenuated ASFV strains affected moMΦ cytokine responses to classical activation (IFN-γ and LPS) (data not shown), suggesting that the synthesis of ASFV viral proteins is required to inhibit macrophage response to external stimuli. ASFV encodes several proteins involved in evasion of host defenses [24], including A238L that inhibits inflammatory responses through NF-kB regulation [24], and this might result in reduced ability to respond to external stimuli. In addition, NH/P68 presented a more pronounced inhibitory effect compared to 22653/14, and this might be linked to its higher replication efficacy early time pi.

Our analyses showed that both attenuated and virulent ASFV strains efficiently infected and replicated in all macrophage subsets tested, with NH/P68 infection resulted in higher replication efficiency early pi. Both strains affected macrophage responses to stimulation with either IFN-γ and LPS or Pam2Cys lipopeptide, and 22653/14 infection did not result in any cytokine release or modulation of MHC class I and II expression. Overall, our results suggest that 22653/14 deploys mechanisms to covertly replicate in porcine macrophages, independently of their activation status, and affects their ability to respond to external stimuli. In contrast, the attenuated NH/P68 presented a higher sensitivity to classical and IFN-α activation, and infection resulted in downregulated expression of MHC class I in all the subsets and release of IL-1α, IL-1β, and IL-18 from moM1. Thus, NH/P68 modulation of macrophage phenotype and function could enhance immune surveillance and promote the development of a protective immune responses. The data generated in this study provides some insight in the complex interaction of ASFV with its target cells, which we hope will aid our understanding of the immunomodulation of host cell responses by ASFV.

## 4. Materials and Methods

### 4.1. Animals

Healthy, ASFV-naïve, cross-bred pigs (*Sus scrofa*), 6-24 months old, were housed at the Experiment Station of IZS of Sardinia (Sassari, Italy). Animal husbandry and handling procedures were performed in accordance with the local ethics committee, in agreement with the Guide of Use of Laboratory Animals issued by the Italian Ministry of Health. The ASFV-negative status of the animals was confirmed by three different laboratory tests: PCR, a commercial ELISA test (Ingezim PPA Compac ^®^, Ingenasa, Madrid, Spain) and an immunoblotting test, as suggested by the Manual of Diagnostic Tests and Vaccines for Terrestrial Animals [25].

### 4.2. Viruses

Several ASFV isolates were used in this study: virulent Sardinian field strains 22653/14 and Nu81.2 (both isolated from naturally infected pigs during ASF outbreaks in Sardinia in 2014 and 1981, respectively) (Exotic Disease Laboratory ASF Virus Archive, IZS of Sardinia, Sassari, Italy), the virulent strains Arm07 and Ken06.Bus (kindly provided by the EU ASF Reference Laboratory CISA-INIA, Madrid, Spain), and the attenuated NH/P68 (kindly provided by the EU ASF Reference Laboratory CISA-INIA, Madrid, Spain). NH/P68 is a non-fatal, non-haemoadsorbing ASFV strain isolated from a chronically infected pig in Portugal in 1968 [22,26]. ASFV strains were propagated in vitro using 25 cm^2^ flask (Corning, NY, USA), by inoculation of sub-confluent monolayers of monocytes/macrophage cultures [25,27]. In brief, leukocytes were cultured two days in RPMI-1640 medium supplemented with 20% (v/v) autologous plasma, 100 U/mL penicillin and 100 μg/mL streptomycin. Then, non-adherent cells were removed, virus suspension was added to the adherent two days old monocytes/macrophage monolayer, and after 2 h of incubation new medium was added. After two or three days at 37 °C in 5% CO_2_, the supernatant was collected and pooled with a freeze-thawed cell lysate. The resultant pool was clarified by centrifugation at 3000× *g* for 15 min, aliquoted and stored at −80 °C. Titers of haemadsorbing 22653/14, Nu81.2, Arm07, Ken06.Bus ASFV strains were obtained by serial dilution of the virus suspension on two days old monocyte/macrophage cultures in 96-well plates followed by haemadsorption [25]. Viral titres of 22653/14 and the non-haemadsorbing NH/P68 were obtained by serial dilution of the virus suspension on monocyte/macrophages in 96-well plates followed by immunofluorescence staining [25]. Viral titres were determined using the Spearman–Kärber formula. Mock-virus supernatants were prepared in an identical manner from uninfected monocyte/macrophage cultures. The attenuated NH/P68 and field strains 22653/14 and Nu81.2 belong to genotype I [6,28] whereas Arm07 and Ken06.Bus belong to genotype II and IX, respectively [28,29].

### 4.3. Generation and Activation of Porcine Monocyte-Derived Macrophages

Porcine leukocytes were obtained from heparinized blood, as previously described [30]. In brief, blood was centrifuged at 700× *g* for 30 min at 4 °C with no breaks, buffy coat was collected and washed in red blood cell lysis buffer (distilled water with 0.5 mM Na EDTA, 310 mM NH4Cl, 24 mM NaHCO3) and cells were re-suspended in RPMI-1640 supplemented with 10% FBS and 100 U/mL penicillin and 100 μg/mL streptomycin (complete RPMI, cRPMI). Macrophage cultures were obtained using petri dishes, as previously described with slight modification [30]. In brief, leucocytes were incubated at 37 °C 5% CO_2_ in petri dishes in cRPMI supplemented with 50 ng/mL of recombinant human M-CSF (hM-CSF) (Thermo Fisher Scientific, Waltham, MA, USA) for seven days. Then leukocytes were removed, adherent cells were detached by gentle scraping with pipette, centrifuged at 200× *g* for 8 min, viable cell counts were obtained using a Countess Automated Cell Counter (Thermo Fisher Scientific). 7–8 × 10^5^ live cells/well were seeded in a 12 well plates (Greiner CELLSTAR, Sigma-Aldrich, Saint Louis, MO, USA) in cRPMI. After seeding, macrophages were incubated at 37 °C 5% CO_2_ for further 24 h: they were left untreated (moMΦ) or stimulated with activators to induce classical or alternative activation, as previously described [10,11]. moMΦ were activated with 100 ng/mL of recombinant porcine IFN-γ (Raybiotech Inc, Norcross, GA, USA) and 100 ng/mL of LPS (Lipopolysaccharide from Escherichia coli 0111:B4; Sigma-Aldrich) to achieve classical activation (moM1) or 20 ng/mL of recombinant porcine IL-4 (R&D Systems, Minneapolis, MN, USA) to achieve alternative activation (moM2) or with 100 U/mL recombinant porcine IFN-α (PBL Assay Science, Piscataway, NJ, USA) (‘moMΦ + IFN−α’). In defined experiments, cells were activated using different concentration of IFN−α (800, 400, 200, 100 U/mL).

### 4.4. ASFV Infection of Macrophage Subsets

Supernatants from macrophage cultures were removed and replaced with ASFV at a MOI of 1. After 90 min incubation at 37 °C 5% CO_2_, virus inoculum was removed, cells were washed with unsupplemented RPMI-1640 medium and fresh cRPMI was added to the wells [3,19]. Cells were incubated at 37 °C 5% CO_2_ and harvested after several time post-infection (pi). Mock-infected controls were included in each experiment. In order to evaluate viral infection, modulation of surface markers and cytokine release in response to ASFV infection, cells were harvested, and culture supernatants were collected at 21 h pi. Supernatants were clarified by centrifugation at 2000× *g* for 3 min and stored at −80 °C until analysed. To evaluate expression of a subset of cytokine genes, cells were infected using a MOI of 1 and collected at 3, 6, 9, 12, 21 h pi. Supernatants were removed and cells were stored at −80 °C until analysed.

### 4.5. Growth Kinetics of ASFV in Macrophage Subsets

Macrophage culture medium was removed and replaced with ASFV at a MOI of 0.01. After 90 min incubation at 37 °C 5% CO_2_, virus inoculum was removed, cells were washed with un-supplemented RPMI-1640 medium and fresh cRPMI was added to the wells. Cells were incubated at 37 °C 5% CO_2_ and culture supernatants were collected at 0, 24, 48, 72 h pi. Supernatants were clarified by centrifugation at 2000× *g* for 3 min and stored at −80 °C until analysed. Levels of infectious virus were determined by titration as described above.

### 4.6. ASFV Effect on Response to M1 Activation or Stimulation with TLR2 Agonist

To evaluate effect of ASFV on M1 activation, moMΦ were infected with NH/P68 or 22653/14 using a MOI of 1, alongside mock-infected controls. After 21 h, media was removed and replaced with cRPMI alone or supplemented with 100 ng/mL of recombinant porcine IFN-γ and 100 ng/mL of LPS (M1 activation), as previously described. After 24 h, supernatants were removed, cleared by centrifugation (2000× *g* for 3 min) and stored at −80 °C until determination of cytokine levels.

To evaluate effect of ASFV to response of moMΦ to this TLR2 agonist, moMΦ were infected with NH/P68 or 22653/14 using a MOI of 1, alongside mock-infected controls. 21 h pi, media was removed and replaced with cRPMI supplemented with 1 μg/mL of a *S*-[2–bis(palmitoyl)-propyl]cysteine (Pam2Cys) lipopeptide (kindly provided by Prof. Bernardo Chessa and Dr. Carla Cacciotto, University of Sassari, Italy), and after 24 h supernatants were removed, cleared by centrifugation (2000× *g* for 3 min) and stored at −80 °C until determination of cytokine levels. Pam2Cys lipopeptide was synthesized based on the 14 amino acids following the cysteine immediately downstream the signal peptide of a *Mycoplasma agalactiae* lipoprotein (P48: CGDKYFKETEVDGV) [31].

### 4.7. DNA Extraction and Real-Time PCR

Viral DNA was extracted from 0.2 mL of cell culture supernatants and finally eluted in 90 μl of RNase-free water, using High Pure PCR Template Preparation Kit, according to the manufacturer’s protocols (Roche, Mannheim, Germany). ASFV genome copies were assessed by real-time PCR as previously described [17,32] using the TaqMan Fast Advanced Master Mix (Applied Biosystems, Waltham, MA, USA), 0.8 µM of sense and anti-sense primers (5′-CTG CTC ATG GTA TCA ATC TTA TCG A-3′and 5′-GAT ACC ACA AGA TCR GCC GT-3′), 0.2 µM of TaqMan probe 5′-[6-carboxy-fluorescein (FAM)]-CCA CGG GAG GAA TAC CAA CCCAGT G-3′-[6-carboxy-tetramethyl-rhodamine (TAMRA)] in a total volume of 25 µL containing 5 µL of extracted DNA. The thermal profile was as follows: a denaturation step at 95 °C for 10′, 40 cycles of denaturation at 95 °C for 15″, then annealing at 58 °C for 60″. Viral copies number present in the 5 μL of the extracted DNA were determined using the plasmid pEX-K4-ASFV-E70p72 (Eurofins Genomics, Louisville, KY, USA), which contains a full length p72 sequence, was used as the template to prepare the standard curve for the real-time PCR assay. For each experiment, a standard curve was prepared by serial dilution (10^−1^ − 10^−7^ copy/μL) of pEX-K4-ASFV-E70p72 template DNA.

### 4.8. Cytofluorometric Analysis

Cytofluorimetric analysis was performed as previously described [6]. In brief, macrophages were harvested from cultures and transferred to wells of a 96-well round-bottom plate. Viability was assessed by staining the cells using a LIVE/DEAD^®^ Fixable Far Red Dead Cell Stain Kit (Thermo Fisher Scientific) for 30 min at 4 °C, and the cells were then washed twice with PBS supplemented with 2% FBS. Expression of surface markers were determined using MHC class II DR (2E9/13; Bio-Rad Antibodies, Kidlington, UK) and MHC class I (JM1E3; Bio-Rad Antibodies) monoclonal antibodies. Their staining was visualized by subsequent staining with RPE-conjugated goat anti-mouse IgG-Fc polyclonal antibody (Thermo Scientific Pierce). Intracellular levels of the late ASFV protein p72 were determined using an anti-p72-FITC antibody (18BG3, Ingenasa). Flow cytometry analysis was performed on a BD FACSCalibur instrument (BD Biosciences, Franklin Lakes, NJ, USA), and at least 5,000 live macrophages were acquired. Analysis of data was performed using Cell Quest Pro Software (BD Biosciences), by gating on viable cells (Live/Dead Fixable Dead Cell Stain negative) in the macrophage population, and then assessing the staining for surface markers and intracellular ASFV p72 protein. Gates for p72 protein were set using the mock-infected controls, whereas gates for surface markers were set using the corresponding isotype controls [6].

### 4.9. Analysis of the Cytokine Levels in Culture Supernatants of Monocyte-derived Macrophage Subsets to ASFV Infection

The simultaneous measurement of IL-1α, IL-1β, IL-6, IL-10, IL-12, IL-18 and TNF-α in macrophage culture supernatants (21 h pi) were performed using Porcine Cytokine/Chemokine Magnetic Bead Panel Multiplex assay (Merck Millipore, Darmstadt, Germany) and a Bioplex MAGPIX Multiplex Reader (Bio-Rad, Hercules, CA, USA), according to the manufacturers’ instructions.

### 4.10. Analysis of Cytokine Gene Expressions

Expression of IL-1β, IL-6, IL-10, IL-18, TNF-α, IL-12p40 genes in ASFV-infected moMΦ at selected time-points (0, 3, 6, 9, 12, 21 h pi) was evaluated. Total RNA was extracted from macrophages using RNeasy Mini Kit (Qiagen, Hilden, Germany), eluted in 50 µl of ultrapure RNase-free water and then 100 ng of purified RNA were used as template for cDNA synthesis, as previously described [33]. The expression of the above genes was determined by RT- qPCR using primer sets previously described (Il-1β, IL-6, IL-18, TNF-α in Razzuoli et al. [33]; IL-12p40 in Shabir et al. [34]; IL-10 in Zanotti et al. [35]) and glyceraldehyde 3-phosphate dehydrogenase (GAPDH) was used as reference gene [33]. EVA Green Real-Time PCR amplification was performed in a CFX96™ Real-Time System after the reverse transcription step, as previously described [33]. Six experiments each using different blood donor pigs were carried out. In each sample, the relative expression of the cytokine genes was calculated using the formula 2−ΔΔCt, where Ct is short for cycle of threshold, ΔCt = Ct (target gene) − Ct (reference gene), and ΔΔCt = ΔCt (ASFV infected sample) − ΔCt (mock-infected sample).

### 4.11. Data Analysis and Statistics

Experiments were performed in technical duplicate (titration, multiplex assay, and qPCR) or triplicate (flow cytometry) and were repeated at least three times with macrophages from different blood donor pigs. Mean data are presented with standard deviations (SD) quoted to indicate the uncertainty around the estimate of the group mean. Graphical and statistical analysis was performed using GraphPad Prism 8.01 (GraphPad Software Inc, La Jolla, CA, USA) and Minitab 2018 (Minitab Inc., Coventry, UK). All data were checked for normality using the Anderson-Darling test and analysed by the parametric unpaired T-test or the non-parametric Mann-Whitney test or Kruskal-Wallis followed by Dunn’s multiple comparison test.

## Supplementary Materials

The following are available online at https://www.mdpi.com/2076-0817/9/3/209/s1, Figure S1: Comparison of the mean fluoresce intensity of p72^+^ porcine monocyte-derived macrophage subsets infected with NH/P68 or 22653/14, Figure S2: Genotype-independent susceptibility of IFN−α−activated moMΦ to virulent ASFV, Figure S3: Gating strategy adopted to analyse ASFV modulation on surface markers.
