# Supplementary material for: Comparison of Macrophage Responses to African Swine Fever Viruses Reveals that the NH/P68 Strain is Associated with Enhanced Sensitivity to Type I IFN and Cytokine Responses from Classically Activated Macrophages
Source: Pathogens. 2020 Mar 12;9(3):209. doi: 10.3390/pathogens9030209 (PMC7157553; doi:10.3390/pathogens9030209)
Supplement: Supplementary file 1 [file pathogens-09-00209-s001.pdf]

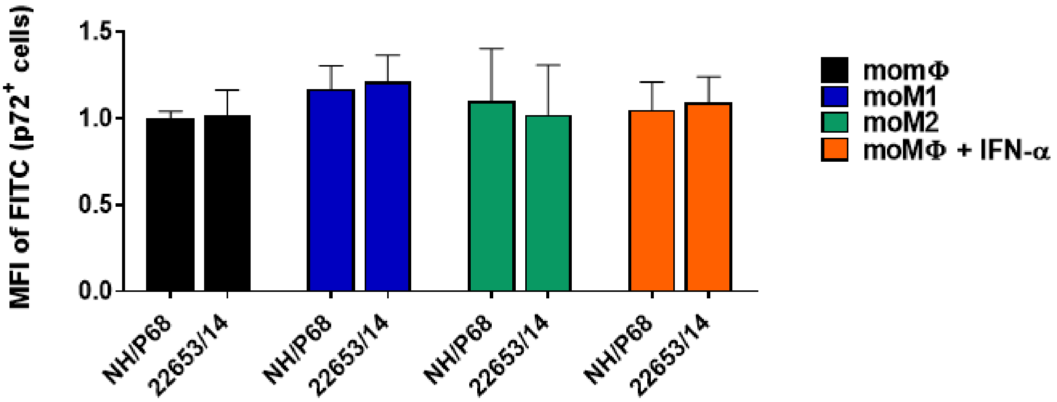

Figure S1. Comparison of the mean fluoresce intensity of p72+ porcine monocyte-derived macrophage subsets infected with NH/P68 or 22653/14. moMΦ, moM1, moM2, IFN-α-activated moMΦ were infected with the low virulence NH/P68 or the virulent 22653/14 ASFV strains using an MOI of 1, alongside mock-infected controls. 21 hours pi the mean fluorescence intensity (geometric mean) of ASFV p72+ cells were assessed. The mean data +/- SD from four independent experiments utilizing different animals are shown. MFI data are presented as fold change relative to the NH/P68-infected moMF. NH/P68 and 22653/14 values were compared using a Mann-Whitney test; ***$p<0.001$, **$p<0.01$, *$p<0.05$.

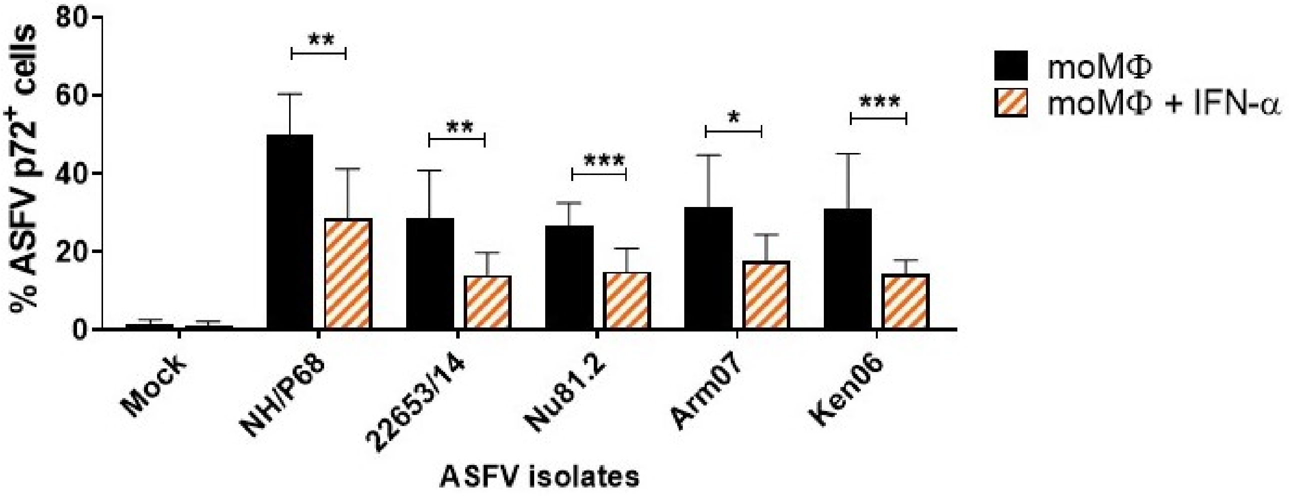

Figure S2. Genotype-independent susceptibility of IFN-α-activated moMΦ to virulent ASFV. moMΦ were left untreated or activated with a high dose of recombinant porcine IFN-α (800 U/ml). After 24 hours, cells were infected with genotype I (NH/P68, 22653/14, NU81.2), genotype II (Arm07) or genotype IX (Ken06.Bus) ASFV strains using a MOI of 1, alongside mock-infected controls. 21 hours pi percentages of ASFV $p72^{+}$ cells were evaluated using flow cytometry. The mean data +/- SD from four independent experiments utilizing different animals are shown. For each strain, values of IFN-α-activated moMΦ were compared to the corresponding un-treated condition (moMΦ), using a Mann-Whitney test; ***$p<0.001$, **$p<0.01$, *$p<0.05$.

.

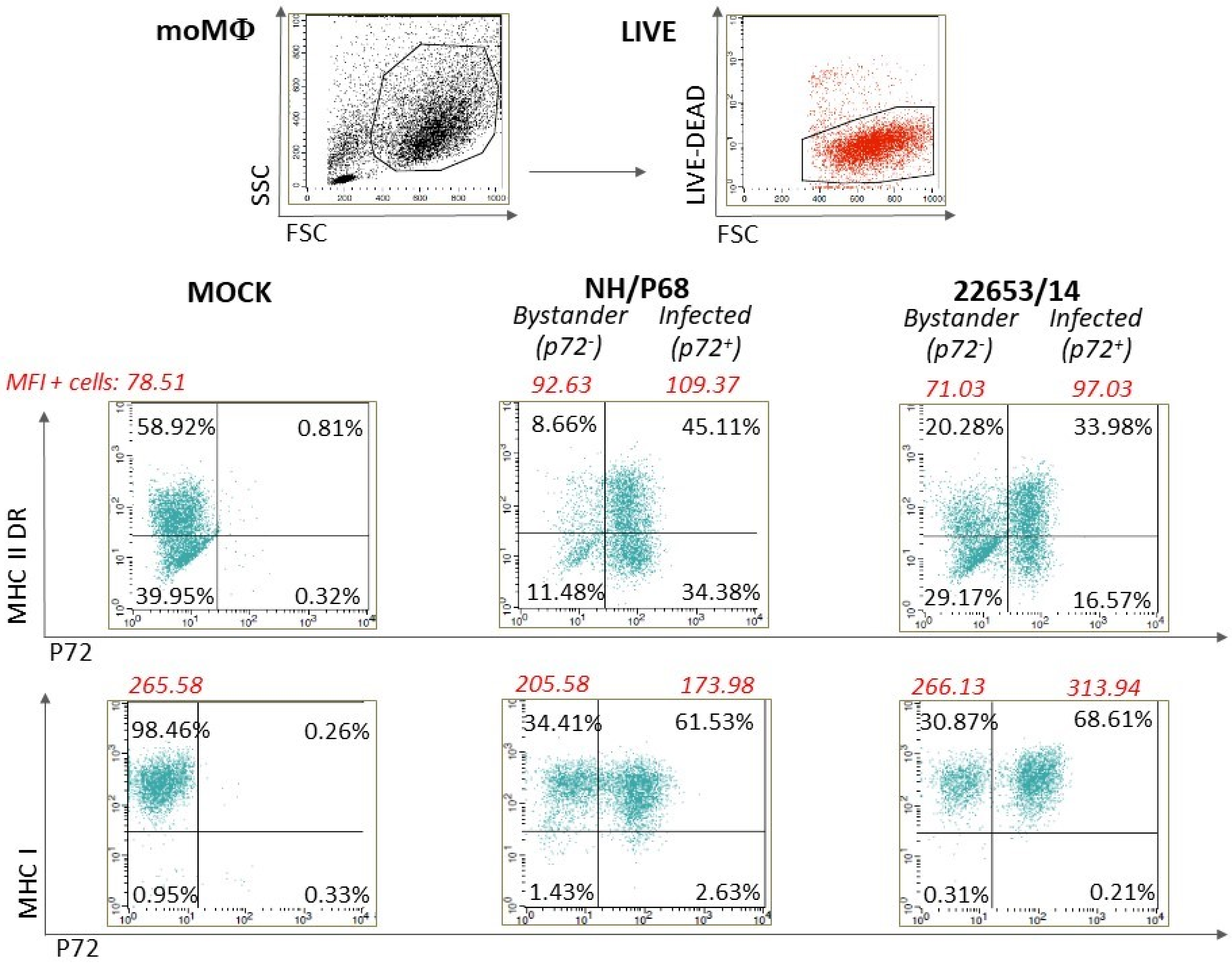

Figure S3. Gating strategy adopted to analyse ASFV modulation on surface markers. On the top, gating strategy used to investigate surface marker expressions and intracellular levels of ASFV late viral protein p72 is displayed. Below, for both MHC class II DR and MHC class I, representative dot plots of mock-infected, NH/P68-infected or 22653/14-infected moMΦ are displayed.
